# Admission Random Blood Glucose, Fasting Blood Glucose, Stress Hyperglycemia Ratio, and Functional Outcomes in Patients With Acute Ischemic Stroke Treated With Intravenous Thrombolysis

**DOI:** 10.3389/fnagi.2022.782282

**Published:** 2022-02-08

**Authors:** Guangyong Chen, Junli Ren, Honghao Huang, Jiamin Shen, Chenguang Yang, Jingyu Hu, Wenjing Pan, Fangyue Sun, Xinbo Zhou, Tian Zeng, Shengqi Li, Dehao Yang, Yiyun Weng

**Affiliations:** ^1^Department of Neurology, The Third Affiliated Hospital of Wenzhou Medical University, Wenzhou, China; ^2^School of the First Clinical Medical Sciences, Wenzhou Medical University, Wenzhou, China; ^3^Department of Neurology, The Second Affiliated Hospital, Zhejiang University School of Medicine, Hangzhou, China; ^4^Department of Neurology, The First Affiliated Hospital of Wenzhou Medical University, Wenzhou, China

**Keywords:** stress hyperglycemia ratio, stroke, intravenous thrombolysis, random blood glucose, fasting blood glucose

## Abstract

**Background:**

Stress hyperglycemia ratio (SHR), calculated as glucose/glycated hemoglobin, has recently been developed for assessing stress hyperglycemia and could provide prognostic information for various diseases. However, calculating SHR using random blood glucose (RBG) drawn on admission or fasting blood glucose (FBG) could lead to different results. This study intends to evaluate the association between SHR and functional outcomes in patients with acute ischemic stroke (AIS) with recombinant tissue plasminogen activator (r-tPA) intravenous thrombolysis.

**Methods:**

Data from 230 patients with AIS following thrombolytic therapy with r-tPA in the Third Affiliated Hospital of Wenzhou Medical University from April 2016 to April 2019 were retrospectively reviewed. SHR1 was defined as [RBG (mmol/L)]/[HbA1c (%)] and SHR2 was defined as [FBG (mmol/L)]/[HbA1c (%)]. The outcomes included early neurological improvement (ENI), poor function defined as a modified Rankin Scale score (mRS) of 3–6, and all-cause death in 3 months. Multivariable logistic regression was performed to estimate the association between SHR and adverse outcomes.

**Results:**

After adjustment for possible confounders, though patients with AIS with higher SHR1 tend to have a higher risk of poor outcome and death and unlikely to develop ENI, these did not reach the statistical significance. In contrast, SHR2 was independently associated with poor functional outcome (per 0.1-point increases: odds ratios (OR) = 1.383 95% CI [1.147–1.668]). Further adjusted for body mass index (BMI), triglyceride-glucose index (TyG), and diabetes slightly strengthen the association between SHR (both 1 and 2) and adverse outcomes. In subgroup analysis, elevated SHR1 is associated with poor functional outcomes (per 0.1-point increases: OR = 1.246 95% CI [1.041–1.492]) in non-diabetic individuals and the association between SHR2 and the poor outcomes was attenuated in non-cardioembolic AIS.

**Conclusion:**

SHR is expected to replace random or fasting glucose concentration as a novel generation of prognostic indicator and a potential therapeutic target.

## Introduction

Acute ischemic stroke (AIS), a pervasive type of stroke, the major therapeutic method, is intravenous or intra-arterial recombinant tissue plasminogen activator (r-tPA) or mechanical endovascular therapies. However, there are inherent risks in terms of the process of thrombolysis using r-tPA, while benefiting eligible patients. That is why it is crucial to find biomarkers that can predict the prognosis of patients with AIS. Previous studies have demonstrated that the poor clinical outcome following thrombolytic therapy with r-tPA for patients with AIS was associated with an elevated random blood glucose (RBG) concentration drawn on admission or an elevated fasting blood glucose (FBG) concentration (Capes et al., 2001), both of which had limitations in distinguishing chronic poor management of background glucose levels and physiological stress response to AIS.

Stress hyperglycemia manifests as transient hyperglycemia in the context of illness with or without known diabetes. Recently, a novel index introduced by Roberts et al. (2015) called stress hyperglycemia ratio (SHR) was applied for assessing stress hyperglycemia. Considering that the glycosylated hemoglobin (HbA1c) was a relatively stable index that could reflect the glucose control of patients with diabetes in the past 3 months, SHR was defined as the admission glucose concentration divided by the estimated average glucose (eAG) concentration derived from the HbA1c (Nathan et al., 2008; Roberts et al., 2015). However, Hempe et al. (2010) pointed out that the term “eAG” should be used carefully for clinical practice due to discrepancies between eAG and self-monitored mean blood glucose. Another definition of SHR using the glucose/HbA1c ratio was more practical and widely applied in many studies (Zhu et al., 2019; Li et al., 2020; Merlino et al., 2020; Yuan et al., 2021).

Previous studies suggested that the elevated SHR was associated with the poor AIS functional outcome following mechanical thrombectomy or intravenous thrombolysis (Chen et al., 2019; Merlino et al., 2020; Ngiam et al., 2020), higher risk of post-AIS hemorrhagic transformation (Merlino et al., 2020; Yuan et al., 2021), and stroke recurrence (Zhu et al., 2019). However, meaningful conclusions from these studies are limited as SHR were calculated using FBG in most of the studies. Compared with FBG, RBG could be obtained in an earlier stage and beneficial for risk stratification and making medical decisions. A large prospective cohort study among the Chinese population suggested that hyperglycemia on admission increased the risk of poor functional outcomes in patients with AIS with intravenous thrombolysis (Lin et al., 2018). Higher RBG on admission is also associated with a low rate of recanalization in individuals with rt-PA intravenous thrombolysis (Saqqur et al., 2015). Admission SHR has been reported to be associated with clinical outcomes in patients presented to the emergency department (Su et al., 2017) and in patients after percutaneous coronary intervention (Yang et al., 2017). The association between admission SHR and AIS prognosis remained unclear. Therefore, the purpose of this study was to explore the relationship between the SHR (calculating using RBG and FBG) and the clinical outcomes after thrombolytic therapy with r-tPA for patients with AIS.

## Materials and Methods

### Study Population

**FIGURE 1:**
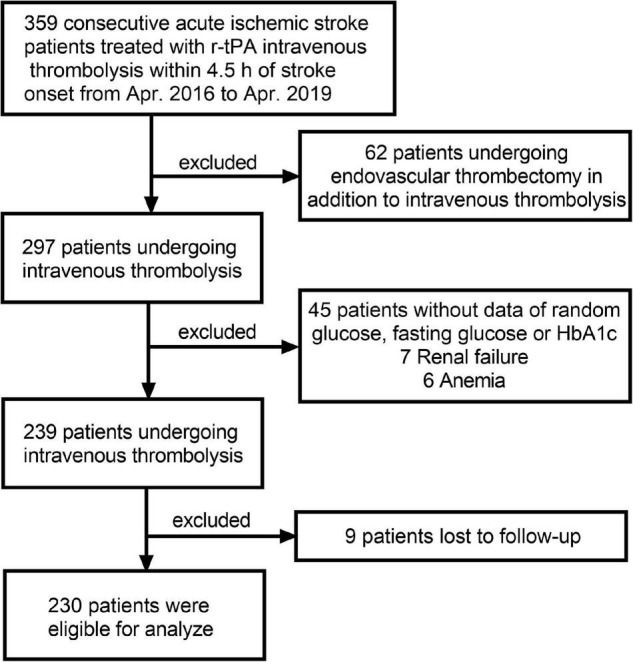
Flow diagram showing the patient selection process.

A total of 359 patients, with a clinical diagnosis of AIS following thrombolytic therapy with r-tPA (0.9 mg/kg to maximum 90 mg, 10% of the dose as a bolus, and the rest by a 60-min infusion) within 4.5 h stroke onset were derived from the Third Affiliated Hospital of Wenzhou Medical University from April 2016 to April 2019. They were excluded for the following exclusion criteria: (1) reception of bridging therapy followed by (2) with conditions affecting the HbA1c level, including renal failure (serum creatinine concentration greater than 180 μmol/L) and anemia (hemoglobin < 100 g/L); (3) with the incomplete RBG, FBG, and HbA1c information; (4) with incomplete follow up data. Finally, 230 cases were included in this analysis (Figure 1). The study was approved by the Ethics Committee of the Third Affiliated Hospital of Wenzhou Medical University and was performed in accordance with the Declaration of Helsinki. There was no requirement for informed consent given the retrospective nature of this study.

### Data Collection

The clinical data of patients with AIS were collected by looking up the electronic medical record. Demographic characteristics [e.g., age, sex, and body mass index (BMI)], medical history (e.g., hypertension, diabetes mellitus, hyperlipidemia, history of stroke, atrial fibrillation, and smoking), laboratory tests including hemoglobin, creatinine, total cholesterol (TC), triglyceride (TG), low-density lipoprotein (LDL), high-density lipoprotein (HDL), and blood pressure measurements were gathered within 24 h admission. RBG was measured on hospital admission. The fasting serum samples within 24 h after admission were drawn during the morning hours after an overnight fast (at least 8 h) to measure FBG. The HbA1c level was tested by using high-performance liquid chromatographic analysis. Stroke severity was evaluated by the National Institutes of Health Stroke Scale (NIHSS) on admission, 24 h after intravenous thrombolysis, and the 7th day. Three-month modified Rankin Scale (mRS) scores after the onset of AIS, collected by two trained physicians on the telephonic interview, were used to evaluate the functional outcome.

### Clinical Assessment

Etiology of patients with AIS was classified based on the Trial of ORG 10172 in Acute Stroke Treatment (TOAST) criteria (Adams et al., 1993) as large artery atherosclerosis (LAA), small artery occlusion (SAO), cardioembolic (CE), other (SOE), and undetermined subtypes (SUE). BMI was calculated as weight (kg) ratio to height squared (m^2^). The triglycerides and glucose (TyG) index, a concise tool with high sensitivity and specificity for identifying insulin resistance, were calculated as ln[fasting TG (mg/dl) × FBG (mg/dl)/2] (Simental-Mendia et al., 2008; Guerrero-Romero et al., 2010). Diabetes status was diagnosed according to a diabetes history and/or an HbA1c level of ≥ 6.5%. SHR was defined as the index of glucose/HbA1c ratio, calculating using the following formula: SHR1 = RBG (mmol/L)/HbA1c (%) while SHR2 = FBG (mmol/L)/HbA1c (%). The primary outcomes included the poor functional outcome, defined as 3-month mRS scores 3–6; the secondary outcomes included early neurological improvement (ENI) defined as a decrease in the NIHSS score by ≥ 4 points in the total score or an NIHSS score 0–1 within 24 h after thrombolysis (Desilles et al., 2013; Gong et al., 2021), and 3-month all-cause mortality.

### Statistical Analysis

We used SPSS Statistics 25.0 software (SPSS Inc., Chicago, IL, United States), MedCalc Statistical Software version 15.2.2 (MedCalc Software bvba, Ostend, Belgium; 2015),^1^ and packages “forestplot” version 2.0.1 and “rms” version 6.2.0 as implemented in R version 4.1.0 (R Foundation for Statistical Computing, Vienna, Austria) for plots and statistical analysis. A two-tailed *p* < 0.05 was considered to be statistically significant.

Continuous variables that met normal distribution were presented with mean ± SD while these not normally distributed were presented with median and interquartile range (IQR). Categorical variables are presented as frequency and percentage. Differences among the two groups were compared through Student’s *t*-test, Mann-Whitney *U*-test, or chi-squared test when appropriate. Receiver operating characteristic (ROC) curves were employed to determine the optimal cutoff values of SHR to distinguish poor functional outcomes. We used a logistic regression model to estimate odds ratios (OR) with 95% CIs for adverse outcomes. The crude model was univariable analysis. In model 1, we adjusted for age and sex, and in model 2, we adjusted for age, sex, and other covariates with a *p*-value < 0.1 in the univariable analysis. Restricted cubic splines (model 2) with 3 knots (at the 10th, 50th, and 90th percentiles) were further plotted to find the association between SHR level and AIS outcome.

We additionally adjusted for BMI, TyG, and diabetes in model 3. However, as this approach might obscure the relationships between these intrinsically linked covariates, subgroup analysis was consequently performed and we tested the statistical significance of covariate category × SHR in the multivariable logistic model to examine the multiplicative interaction. It should be noticed that though patients without information of HbA1c, RBG, FBG, and functional outcomes were already excluded, missing data exist in other covariates. In addition, exist of patients with too high or too low SHR levels might affect the robustness of the regression model. In the main analysis, we replaced the small number of missing data with a mean value of the entire cohort. In the first sensitivity analysis, patients with missing data, bottom 5% SHR, or top 5% SHR were omitted. In the second sensitivity analysis, another definition of SHR suggested by Roberts et al. (2015) was employed: SHR = [RBG or FBG (mmol/L)]/[(1.59 × HbA1c)−2.59].

## Results

### Characteristics of Study Samples

A total of 230 patients with AIS were included in this study, with a mean age of 68 years, and 143 (62.1%) of them were men and 87 (37.9%) were women. The median NIHSS score on admission was 7. A total of 72 (31.3%) patients developed poor functional outcomes, 25 patients (10.87%) dead during the follow-up time, and 82 (35.65%) patients had ENI. The clinical characteristics of patients with AIS are presented in Table 1. In comparison with patents with good functional outcome, those with poor functional outcomes were significantly older (74.67 ± 10.82 vs. 65.32 ± 12.03, *p* < 0.001), had lower BMI (22.50 ± 3.40 vs. 23.49 ± 3.33, *p* = 0.039), higher FBG, (7.04 ± 3.06 vs. 5.78 ± 2.00, *p* = 0.002), higher SHR2 (1.04 ± 0.26 vs. 0.88 ± 0.18, *p* < 0.001), higher NIHSS on admission (13 [8–18] vs. 6 [4–9], *p* < 0.001), and 24 h (12 [7–17] vs. 4 [2–6], *p* < 0.001), and contained a significantly smaller proportion of smoking (11.11 vs. 30.38%, *p* = 0.002), higher proportion of prior stroke (23.61 vs. 7.59%, *p* = 0.001), and CE subtype.

**TABLE 1:** Characteristics of patients with AIS with good (mRS 0–2) vs. poor functional outcomes (mRS 3–6).

Characteristics	Function outcomes		
	mRS 0–2 (*n* = 158)	mRS 3–6 (*n* = 72)	*p-*value
**Demographic data**			
Age (years)	65.32 ± 12.03	74.67 ± 10.82	<0.001
Sex (male, *n*.%)	104 (66.82)	39 (54.17)	0.115
BMI (kg/m^2^)	23.49 ± 3.33	22.50 ± 3.40	0.039
**Stroke risk factors**			
Current smoking *n* (%)	48 (30.38)	8 (11.11)	0.002
Hypertension *n* (%)	93 (58.86)	47 (65.28)	0.355
Diabetes *n* (%)	53 (33.54)	23 (31.94)	0.811
Hyperlipidemia *n* (%)	13 (8.23)	12 (16.67)	0.057
Atrial fibrillation *n* (%)	30 (18.99)	22 (30.56)	0.052
Prior stroke *n* (%)	12 (7.59)	17 (23.61)	0.001
**Laboratory data**			
Hemoglobin (g/L)	134.47 ± 13.46	131.19 ± 16.63	0.114
Creatinine (umol/L)	70.18 ± 16.92	71.53 ± 16.21	0.570
TC (mmol/L)	4.56 ± 1.03	4.78 ± 1.08	0.382
TG (mmol/L)	1.57 ± 1.35	1.32 ± 0.85	0.156
HDL (mmol/L)	1.12 ± 0.27	1.13 ± 0.26	0.670
LDL (mmol/L)	2.91 ± 0.91	3.11 ± 0.92	0.112
HbA1c (%)	6.45 ± 1.16	6.65 ± 1.71	0.309
RBG (mmol/L)	7.93 ± 3.22	8.71 ± 4.05	0.116
FBG (mmol/L)	5.78 ± 2.00	7.04 ± 3.06	0.002
TyG	8.66 ± 0.70	8.71 ± 0.64	0.628
SHR1	1.21 ± 0.31	1.28 ± 0.33	0.098
SHR2	0.88 ± 0.18	1.04 ± 0.26	<0.001
**Clinical data**			
SBP (mmHg)	159.01 ± 24.01	165.53 ± 24.76	0.060
DBP (mmHg)	90.11 ± 15.54	88.56 ± 14.30	0.472
DNT (minute)	55 (45–72)	61 (46–85)	0.131
ONT (minute)	150 (125–196)	172 (138–209)	0.085
NIHSS at admission	6 (4–9)	13 (8–18)	<0.001
NIHSS at 24 h	4 (2–6)	12 (7–17)	<0.001
**Stroke subtype, *n* (%)**			**<0.001**
CE	48 (30.38)	38 (52.78)	
LAA	60 (37.97)	27 (37.50)	
SAO	32 (20.25)	1 (1.39)	
SOE/SUE	18 (11.39)	6 (8.33)	

*BMI, body mass index; TC, total cholesterol; TG, triglyceride; LDL, low-density lipoprotein; HDL, high-density lipoprotein; RBG, random blood glucose; FBG, fasting blood glucose; TyG, triglyceride-glucose index; SHR, stress hyperglycemia ratio; SBP, systolic blood pressure; DBP, diastolic blood pressure; DNT, door to needle time; ONT, onset to needle time; NIHSS, National Institute of Health Stroke Scale; LAA, large artery atherosclerosis; CE, cardioembolism; SAO, small-artery occlusion; SOE, stroke of other determined etiology; SUE, stroke of undetermined etiology.*

### Predictive Values of Glucose and Stress Hyperglycemia Ratio for Poor Functional Outcomes

The ROC curve analysis was employed to determine the predictive value of RBG, FBG, SHR1, and SHR2 for poor functional outcomes (Supplementary Figure 1). Compared with the area under the curve (AUC) of RBG (AUC = 0.556) or FBG (AUC = 0.673) alone, the AUC for SHR1 (AUC = 0.562) or SHR2 (AUC = 0.718) increased by 0.006 or 0.045, respectively. The optimal cutoff values of SHR1 to distinguish poor functional outcomes were 1.35 with 38.89 sensitivity and 78.48 specificity, while SHR2 with a value of 0.92 or more was a fair predictor of poor functional outcomes with 70.83 sensitivity and 72.78 specificity. Characteristics of patients stratified by SHR cutoff values are shown in Supplementary Table 1.

### The Correlation Between Stress Hyperglycemia Ratio and the Primary Outcomes

**FIGURE 2:**
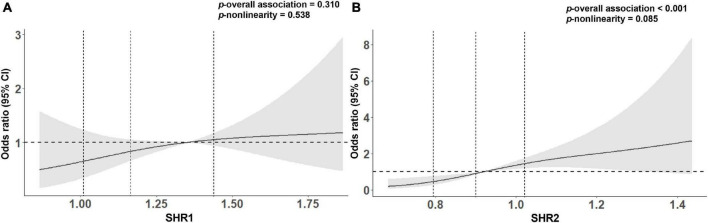
Association between **(A)** SHR1 defined as [admission random blood glucose (RBG) (mmol/L)]/[HbA1c (%)], **(B)** SHR2 defined as [fasting blood glucose (FBG) (mmol/L)]/[HbA1c (%)] and poor clinical outcome on 3-month using restricted cubic splines with 3 knots (at the 10th, 50th, and 90th percentiles). The model was adjusted for age, sex, current smoking, hyperlipidemia, atrial fibrillation, prior stroke, systolic blood pressure (SBP), and NIHSS at admission. The solid line indicates the odds ratio while the shadow indicates 95% CIs. The vertical dashed lines indicate the 1st, 2nd, and 3rd quartiles of SHR. The horizontal dashed line is the reference line (odds ratio = 1). The reference of SHR1 was 1.35, and the reference of SHR2 was 0.92.

We carried both univariate and multivariate logistic regression analyses for the purpose of obtaining a deeper appreciation of the association between SHR levels and the poor functional outcome (Table 2). Although the risk of poor functional outcomes was positively correlated with SHR1 levels, results did not reach statistical significance when SHR1 was calculated as a continuous variable or categorized as quarters. In contrast, the higher SHR2 level was associated with an increasing risk of 3-month poor outcome (Q4 vs. Q1: OR = 9.333, 95% CI [3.445–25.287]; per 0.1 point increase: OR = 1.389, 95% CI [1.202–1.605]) in the crude model without any adjustment. The association between SHR2 and the risk of poor outcome was still significant after adjusting for age and sex in model 1. In model 2, we further adjusted for current smoking, hyperlipidemia, atrial fibrillation, prior stroke, SBP, and NIHSS at admission. SHR2 levels remained a striking predictor of poor outcome in patients with AIS with OR of 10.092 (95% CI, 2.863–35.575, *p* < 0.001) in Q4 and OR of 1.383 (95% CI, 1.147–1.668, *p* = 0.001) per 0.1-point increase. The association between SHR2 and the poor functional outcome was even strengthened after additionally adjusted for BMI, diabetes, and TyG in model 3. To further investigate the correlation between SHR levels and 3-month poor functional outcome, the restricted cubic spline regression of model 2 with 3 knots (at the 10th, 50th, and 90th percentiles) is visualized in Figure 2. Elevated SHR was associated with an increased risk of poor outcome, and a linear association was observed.

**TABLE 2:** Univariate and multivariate logistic regression analyses for 3-month poor functional outcomes.

Variables	Crude Model		Model 1		Model 2		Model 3	
	OR (95% CI)	*p*-value	OR (95% CI)	*p*-value	OR (95% CI)	*p*-value	OR (95% CI)	*p*-value
SHR1 Q1 (< 1.02)	Ref.		Ref.		Ref.		Ref.	
SHR1 Q2 (1.02–1.15)	0.712 (0.315–1.607)	0.413	0.484 (0.195–1.202)	0.118	0.684 (0.227–2.057)	0.499	0.725 (0.231–2.220)	0.574
SHR1 Q3 (1.15–1.44)	0.785 (0.352–1.752)	0.555	0.594 (0.251–1.409)	0.237	1.001 (0.335–2.821)	0.999	1.036 (0.359–2.986)	0.938
SHR1 Q4 (> 1.44)	1.983 (0.937–4.195)	0.073	1.786 (0.785–4.063)	0.167	2.084 (0.794–5.472)	0.136	2.184 (0.792–6.022)	0.131
SHR1 (> 1.35 vs. ≤ 1.35)	2.321 (1.265–4.259)	0.007	2.332 (1.202–4.527)	0.012	2.321 (1.072–5.025)	0.033	2.424 (1.071–5.489)	0.034
SHR1 (per 0.1–point increase)	1.075 (0.986–1.171)	0.100	1.066 (0.971–1.171)	0.178	1.080 (0.971–1.201)	0.154	1.090 (0.970–1.226)	0.147
SHR2 Q1 (< 0.79)	Ref.		Ref.		Ref.		Ref.	
SHR2 Q2 (0.79–0.89)	3.039 (1.079–8.560)	0.035	2.632 (0.896–7.735)	0.078	3.312 (0.935–11.731)	0.063	3.644 (1.014–13.094)	0.048
SHR2 Q3 (0.89–1.03)	7.292 (2.706–19.648)	<0.001	7.301 (2.567–20.762)	<0.001	7.956 (2.273–27.843)	0.001	9.552 (2.611–34.946)	0.001
SHR2 Q4 (> 1.03)	9.333 (3.445–25.287)	<0.001	9.883 (3.442–28.380)	<0.001	10.092 (2.863–35.575)	<0.001	15.205 (3.608–64.085)	<0.001
SHR2 (> 0.92 vs. ≤ 0.92)	6.078 (3.297–11.206)	<0.001	6.550 (3.350–12.807)	<0.001	5.554 (2.522–12.232)	<0.001	7.075 (2.949–16.973)	<0.001
SHR2 (per 0.1–point increase)	1.389 (1.202–1.605)	<0.001	1.452 (1.232–1.712)	<0.001	1.383 (1.147–1.668)	0.001	1.536 (1.212–1.946)	<0.001

*Model 1, adjusted for age and sex.*

*Model 2, adjusted for age, sex, current smoking, hyperlipidemia, atrial fibrillation, prior stroke, SBP, and NIHSS at admission.*

*Model 3, adjusted for covariates from Model 2 and further adjusted for BMI, diabetes, and TyG.*

*The optimal cutoff values of SHR1 (1.35) and SHR2 (0.92) to predict poor functional outcomes were determined using ROC curves.*

*SHR, stress hyperglycemia ratio; SBP, systolic blood pressure; BMI, body mass index; TyG, triglyceride-glucose index; ROC, receiver operating characteristic.*

### Results of Sensitivity and Subgroup Analysis for the Primary Outcome

**FIGURE 3:**
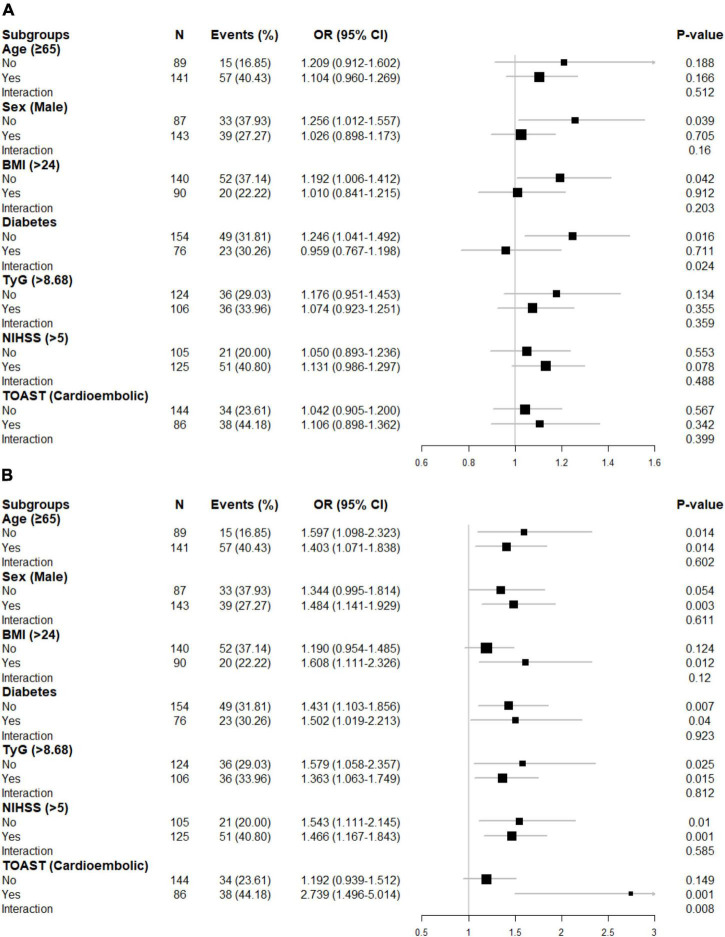
Subgroup analyses for the risk of poor functional outcome by **(A)** SHR1 defined as [admission RBG (mmol/L)]/[HbA1c (%)], **(B)** SHR2 defined as [FBG (mmol/L)]/[HbA1c (%)]. The above model adjusted for age, sex, current smoking, hyperlipidemia, atrial fibrillation, prior stroke, systolic blood pressure (SBP), and NIHSS at admission. In each case, the model is not adjusted for the stratification variable. BMI, body mass index; TyG, triglyceride-glucose index; NIHSS, National Institutes of Health Stroke Scale score; TOAST, Trial of Org 10172 in Acute Stroke Treatment.

In the first sensitivity analysis, 6 patients without complete lipid information and 24 patients with bottom/top 5% SHR1 among the 230 patients were excluded to analyze the association between SHR1 and poor functional outcomes. A similar approach was adopted to analyze the association between SHR2 and poor functional outcomes. Strikingly, the association between SHR1 and poor function seems more apparent in the first sensitivity analysis compared with the main analysis (Table 3). Besides, the second sensitivity analysis using another definition of SHR rendered largely similar results as the main analysis (Supplementary Table 2). In subgroup analysis, we surprisingly found that diabetes might alter the correlation between SHR1 and the primary outcomes (Figure 3). SHR1 was independently associated with poor functional outcomes in non-diabetic AIS (OR = 1.246, 95% CI [1.041–1.492], *p* = 0.016). Furthermore, the association between SHR2 and poor functional outcomes was attenuated in non-cardioembolic AIS.

**TABLE 3:** Univariate and multivariate logistic regression analysis for 3-month poor functional outcome in sensitivity analysis.

Variables	Crude model		Model 1		Model 2		Model 3	
	OR (95% CI)	*p*-value	OR (95% CI)	*p*-value	OR (95% CI)	*p*-value	OR (95% CI)	*p*-value
SHR1 Q1 (< 1.02)	Ref.		Ref.		Ref.		Ref.	
SHR1 Q2 (1.02–1.15)	0.838 (0.349–2.012)	0.692	0.582 (0.222–1.527)	0.272	0.708 (0.212–2.356)	0.573	0.765 (0.224–2.611)	0.669
SHR1 Q3 (1.15–1.44)	0.902 (0.380–2.141)	0.816	0.675 (0.268–1.699)	0.404	1.096 (0.352–3.411)	0.874	1.179 (0.366–3.795)	0.782
SHR1 Q4 (> 1.44)	2.921 (1.219–6.998)	0.016	2.562 (0.994–6.599)	0.051	2.789 (0.898–8.663)	0.076	2.879 (0.903–9.184)	0.074
SHR1 (> 1.35 vs. ≤ 1.35)	3.000 (1.528–5.889)	0.001	2.955 (1.427–6.116)	0.004	2.922 (1.220–6.998)	0.016	2.934 (1.198–7.189)	0.019
SHR1 (per 0.1-point increase)	1.179 (1.045–1.329)	0.007	1.173 (1.019–1.337)	0.017	1.200 (1.027–1.402)	0.021	1.203 (1.026–1.411)	0.023
SHR2 Q1 (< 0.79)	Ref.		Ref.		Ref.		Ref.	
SHR2 Q2 (0.79–0.89)	3.833 (1.169–12.567)	0.027	3.012 (0.884–10.258)	0.078	4.772 (1.035–21.994)	0.045	5.311 (1.115–25.294)	0.036
SHR2 Q3 (0.89–1.03)	9.274 (2.938–29.276)	<0.001	8.168 (2.478–26.922)	0.001	10.845 (2.377–49.479)	0.002	13.609 (2.807–65.982)	0.001
SHR2 Q4 (> 1.03)	9.409 (2.844–31.134)	<0.001	8.201 (2.363–28.457)	0.001	10.204 (2.134–48.784)	0.004	16.976 (3.000–96.054)	0.001
SHR2 (> 0.92 vs. ≤ 0.92)	5.557 (2.897–10.657)	<0.001	5.475 (2.718–11.209)	<0.001	4.682 (1.997–10.977)	<0.001	6.325 (2.524–15.850)	<0.001
SHR2 (per 0.1-point increase)	1.560 (1.260–1.932)	<0.001	1.576 (1.246–1.995)	<0.001	1.509 (1.145–1.987)	0.003	1.770 (1.268–2.471)	0.001

*Model 1, adjusted for age and sex.*

*Model 2, adjusted for age, sex, current smoking, hyperlipidemia, atrial fibrillation, prior stroke, systolic blood pressure (SBP), and NIHSS at admission.*

*Model 3, adjusted for covariates from Model 2 and further adjusted for body mass index (BMI), diabetes, and triglyceride-glucose index (TyG).*

*In the sensitivity analysis, we adopted the previously established groups in Table 2 and excluded participants with top 5% SHR, bottom 5% SHR, or without lipid data.*

*SHR, stress hyperglycemia ratio.*

### The Correlation Between Stress Hyperglycemia Ratio and the Secondary Outcomes

In the univariate analysis, both SHR1 (Q4 vs. Q1: OR = 4.773, 95% CI [1.242–18.345]; per 0.1 point increase: OR = 1.129, 95% CI [1.005–1.269]) and SHR 2 (Q4 vs. Q1: OR = 17.429, 95% CI [2.183–139.159]; per 0.1 point increase: OR = 1.321, 95% CI [1.129–1.547]) were associated with 3-month mortality. However, neither SHR1 nor SHR2 remained significantly associated with mortality in the multivariate-adjusted models (Supplementary Table 3). In addition, no association between SHR and ENI was observed in this study (Supplementary Table 4).

## Discussion

In this retrospective observational cohort study, we explored the association between SHR and the clinical outcomes in patients with AIS with r-tPA intravenous thrombolysis. The main findings were as follows: (1) SHR1 (calculating as RBG/HbA1c ratio) had a higher predictive value for 3-month poor functional outcomes than RBG while SHR2 (calculating as FBG/HbA1c ratio) had a higher predictive value for 3-month poor functional outcomes than FBG; (2) no association between SHR1 and adverse outcomes (poor functional outcome, mortality, and ENI) was found in the main analysis, but SHR1 was independently associated with poor functional outcomes in non-diabetic AIS; (3) higher SHR2 was independently associated with poor functional outcomes at 3 months but not with mortality or ENI; (4) the association between SHR2 and poor functional outcomes was attenuated in non-cardioembolic AIS.

Although a line of evidence suggested that hyperglycemia is associated with worse outcomes in patients with AIS (Desilles et al., 2013; Hafez et al., 2014), the mechanisms of the association between an increased SHR and poor outcome following thrombolytic therapy with r-tPA for patients with AIS were seemingly unclear. Considering most of the patients with non-CE were LAA, this study suggested that the presence of diabetes and atherosclerosis further complicated the association between SHR and AIS outcomes. There were several possible explanations for this phenomenon. (1) Stress hyperglycemia, a stress response due to abnormal regulation of the neurohumoral endocrine system, can result in a cycle of excessive hepatic glucose production and insulin resistance, contributing to the increased blood glucose (Salmasi et al., 2005; Dungan et al., 2009). Not only does the increased blood glucose reduce the fibrinolytic activity of r-tPA, inhibiting the dissolution of venous thrombosis, but also changes the permeability of the blood barrier, leading to cerebral edema (Dietrich et al., 1993; Pandolfi et al., 2001; Dua et al., 2010). However, chronic hyperglycemia might make the neuroendocrine system less sensitive during the acute phase of stroke through downregulation of glucose transporters (Dungan et al., 2009), and consequently alter the association between stress hyperglycemia and stroke outcomes; (2) stress hyperglycemia may give rise to reperfusion injury after r-tPA intravenous thrombolysis by increasing oxidative stress and inflammation (Luitse et al., 2012; Koracevic, 2016). Increased expression of the endothelial adhesion molecules was observed in hyperglycemia status (Omi et al., 2002). Besides, some stress reaction followed stroke may lead to the neuronal expression of monomeric C-reactive protein, which act as a sensor for systemic inflammation and played an important role in the late degeneration of brain tissues (Slevin et al., 2020); (3) patients with AIS are mostly associated with lipid metabolism disorders, increased LDL, and increased blood lipids, which could cause endothelial cell damage and aggravate inflammatory and stress responses. Under the high blood sugar state, the glycated LDL is swallowed by macrophages and transformed into foam cells, which adhere to the blood vessel wall, accelerating the formation of atherosclerosis and cerebrovascular disease complications, affecting prognosis.

In previous studies, scholars focused on the prognostic effect of blood glucose for patients with AIS. Early blood glucose management in AIS was also important in routine clinical practice. Early blood glucose management for hyperglycemia was recommended to be initiated at the first-hour post-AIS (preferably within 12 h), last for more than 48 h, and achieve blood glucose levels in a range of 140–180 mg/dl (Palaiodimou et al., 2019; Powers et al., 2019). However, the random or fasting glucose concentration has the disadvantage in distinguishing between stress hyperglycemia and chronic high background glucose concentration. On the contrary, SHR is a relative indicator that can be quantitatively evaluated for hyperglycemia. Therefore, SHR is expected to replace random or fasting glucose concentration as a novel generation of predictive indicator and a potential therapeutic target.

Definitions of stress hyperglycemia were different among previous studies. The time and method to draw the blood sample were also inconsistent. In addition, patients with bridging therapy after intravenous thrombolysis were not excluded in some studies. This study adopted the two widely used definitions of SHR (refer to the “Materials and Methods” section) and evaluated the stress hyperglycemia using RBG and FBG. Besides, this study demonstrated that the correlation between SHR and AIS functional outcomes might be altered in different diabetes statuses and TOAST subtypes. This study also inevitably had several limitations. There was no way to trace causality for the reason that this study was a retrospective observational cohort study. Our research was designed to collect data only from one hospital, so the sample size was limited, which may result in the selection bias. Although the sensitivity analysis showed that the association between SHR2 and AIS functional outcomes was robust, the association between SHR1 and AIS functional outcomes may underestimate in the main analysis. In addition, many possible mechanisms on the relationship of SHR and prognosis in patients with AIS, especially the early control of hyperglycemia, have not been considered. Thus, experimental studies and future prospective trials should be carried out to support our point of view.

## Conclusion

This study found that SHR (calculating using RBG) was independently associated with poor functional outcomes in patients with non-diabetic AIS with the treatment of intravenous thrombolysis. Patients with AIS with elevated SHR (calculating using FBG) tend to have higher odds of poor functional outcomes. Instead of RBG or FBG, whether SHR could be a novel target for early intervention merit attention in future research.

## Data Availability Statement

The raw data supporting the conclusions of this article will be made available by the authors, without undue reservation.

## Ethics Statement

The studies involving human participants were reviewed and approved by Ethics Committee of the Third Affiliated Hospital of Wenzhou Medical University. Written informed consent for participation was not required for this study in accordance with the national legislation and the institutional requirements.

## Author Contributions

DY and YW conceptualized the study and supervised the study. GC, JR, HH, JS, CY, JH, WP, FS, XZ, TZ, and SL contributed to the acquisition of data. GC, JR, HH, and JS performed the statistical analysis and interpreted data. GC, JR, and HH prepared the manuscript. DY, YW, GC, JR, HH, JS, CY, JH, WP, FS, XZ, TZ, and SL revised the manuscript. All authors approved the protocol.

## Conflict of Interest

The authors declare that the research was conducted in the absence of any commercial or financial relationships that could be construed as a potential conflict of interest.

## Publisher’s Note

All claims expressed in this article are solely those of the authors and do not necessarily represent those of their affiliated organizations, or those of the publisher, the editors and the reviewers. Any product that may be evaluated in this article, or claim that may be made by its manufacturer, is not guaranteed or endorsed by the publisher.

## Footnotes

1
http://www.medcalc.org
