# Supplementary material for: Admission Random Blood Glucose, Fasting Blood Glucose, Stress Hyperglycemia Ratio, and Functional Outcomes in Patients With Acute Ischemic Stroke Treated With Intravenous Thrombolysis
Source: Front Aging Neurosci. 2022 Feb 8;14:782282. doi: 10.3389/fnagi.2022.782282 (PMC8861349; doi:10.3389/fnagi.2022.782282)
Supplement: Supplementary file 1 [file Data_Sheet_1.pdf]

Table S1. Patient characteristics dichotomized according to stress hyperglycemia ratio.

Characteristics	RBG to HbA1c ratio (SHR1)			FBG to HbA1c ratio (SHR2)		
	≤ 1.35 (n = 168)	> 1.35 (n = 62)	*p* value	≤ 0.92 (n = 137)	> 0.92 (n = 93)	*p* value
Demographic data						
Age, (years)	67.80 ± 12.24	69.45 ± 12.93	0.375	67.44 ± 11.98	69.44 ± 13.02	0.233
Sex, (male, n.%)	104 (61.90)	39 (62.90)	0.890	90 (65.69)	53 (56.99)	0.182
BMI, (kg/m^2)	23.10 ± 3.32	23.39 ± 3.56	0.572	22.99 ± 3.23	23.46 ± 3.59	0.297
Stroke risk factors						
Current smoking, n (%)	44 (26.19)	12 (19.35)	0.284	40 (29.20)	16 (17.20)	0.038
Hypertension, n (%)	98 (58.33)	42 (67.74)	0.195	76 (55.47)	64 (68.82)	0.042
Diabetes, n (%)	42 (25.00)	34 (54.84)	< 0.001	33 (24.09)	43 (46.24)	< 0.001
Hyperlipidemia, n (%)	13 (7.74)	12 (19.35)	0.012	10 (7.30)	15 (16.13)	0.035
Atrial fibrillation, n (%)	34 (20.24)	18 (29.03)	0.157	26 (18.98)	26 (27.96)	0.110
Prior stroke, n (%)	22 (13.10)	7 (11.29)	0.714	18 (13.14)	11 (11.83)	0.769
Laboratory data						
Hemoglobin (g/L)	133.61 ± 14.77	132.98 ± 14.11	0.772	133.39 ± 13.69	133.52 ± 15.84	0.950
Creatinine (umol/L)	70.36 ± 15.91	71.26 ± 18.72	0.717	70.73 ± 17.69	70.41 ± 15.16	0.886
TC, (mmol/L)	4.68 ± 1.02	4.74 ± 1.12	0.692	4.59 ± 1.05	4.84 ± 1.02	0.068
TG, (mmol/L)	1.42 ± 1.13	1.71 ± 1.42	0.098	1.37 ± 0.76	1.66 ± 1.67	0.124
HDL, (mmol/L)	1.13 ± 0.26	1.11 ± 0.30	0.621	1.11 ± 0.27	1.14 ± 0.27	0.384
LDL, (mmol/L)	2.98 ± 0.89	2.94 ± 1.00	0.779	2.91 ± 0.93	3.06 ± 0.90	0.240
HbA1c (%)	6.28 ± 0.92	7.16 ± 2.00	< 0.001	6.27 ± 1.04	6.88 ± 1.65	0.002
RBG, (mmol/L)	6.71 ± 1.47	12.12 ± 4.31	< 0.001	7.25 ± 2.69	9.52 ± 4.11	< 0.001
FBG, (mmol/L)	5.67 ± 1.93	7.54 ± 3.12	< 0.001	5.01 ± 1.06	7.89 ± 2.88	< 0.001
TyG	8.57 ± 0.60	8.97 ± 0.78	0.001	8.48 ± 0.53	8.98 ± 0.75	< 0.001
Clinical data						
SBP, (mmHg)	161.24 ± 24.28	160.52 ± 24.85	0.841	157.20 ± 23.27	166.71 ± 25.00	0.004
DBP, (mmHg)	89.77 ± 14.77	89.21 ± 16.25	0.803	87.62 ± 14.74	92.57 ± 15.33	0.015
DNT, (minute)	55 (45-74)	60 (45-77)	0.296	54 (44-73)	60 (49-84)	0.048
ONT, (minute)	155 (126-204)	169 (130-192)	0.755	153 (126-200)	165 (169-202)	0.861
NIHSS at admission	7 (4-11)	8 (5-14)	0.074	6 (4-10)	9 (6-16)	< 0.001
NIHSS at 24h	4 (2-9)	6 (3-11)	0.137	4 (2-7)	9 (4-14)	< 0.001
Stroke subtype, n (%)			0.338			0.567
CE	66 (39.29)	20 (32.26)		48 (35.04)	38 (40.86)	
LAA	65 (38.69)	22 (35.49)		51 (37.23	36 (38.71)	
SAO	20 (11.90)	13 (20.97)		23 (16.79)	10 (10.76)	
SOE/SUE	17 (10.12)	7 (11.29)		15 (10.95)	9 (9.68)	

Abbreviations: BMI body mass index, TC total cholesterol, TG triglyceride, LDL low-density lipoprotein, HDL high-density lipoprotein, RBG random blood glucose, FBG fasting blood glucose, TyG triglyceride-glucose index, SHR Stress hyperglycemia ratio, SBP systolic blood pressure, DBP diastolic blood pressure, DNT Door to needle time, ONT Onset to needle time, NIHSS National Institute of Health Stroke Scale, LAA largeartery atherosclerosis, CE cardioembolism, SAO small-artery occlusion, SOE stroke of other determined etiology, SUE stroke of undetermined etiology,

Table S2. Univariate and multivariate logistic regression analysis for 3-month poor function outcomes using another definition of stress hyperglycemia ratio.

Variables	Crude Model		Model 1		Model 2		Model 3	
	OR (95% CI)	*p* value	OR (95% CI)	*p* value	OR (95% CI)	*p* value	OR (95% CI)	*p* value
SHR1 Q1 (< 0.86)	Ref.		Ref.		Ref.		Ref.	
SHR1 Q2 (0.86-0.97)	0.919 (0.397-2.124)	0.843	0.792 (0.302-1.965)	0.616	1.427 (0.470-4.328)	0.530	1.487 (0.486-4.546)	0.487
SHR1 Q3 (0.97-1.20)	0.992 (0.445-2.211)	0.984	0.838 (0.357-1.967)	0.685	1.526 (0.512-4.555)	0.448	1.512 (0.525-4.707)	0.419
SHR1 Q4 (> 1.20)	2.117 (0.970-4.620)	0.060	1.888 (0.612-4.393)	0.140	2.656 (0.937-7.533)	0.066	2.721 (0.932-7.946)	0.067
SHR1 (per 0.1-point increase)	1.099 (0.986-1.225)	0.089	1.089 (0.969-1.224)	0.153	1.122 (0.981-1.285)	0.094	1.125 (0.978-1.295)	0.099
SHR2 Q1 (< 0.66)	Ref.		Ref.		Ref.		Ref.	
SHR2 Q2 (0.66-0.75)	0.958 (0.332-2.767)	0.937	0.877 (0.289-2.657)	0.816	1.081 (0.302-3.865)	0.905	1.105 (0.307-3.391)	0.879
SHR2 Q3 (0.75-0.88)	3.833 (1.559-9.428)	0.003	4.085 (1.563-10.681)	0.004	4.570 (1.515-13.786)	0.007	4.643 (1.526-14.125)	0.007
SHR2 Q4 (> 0.88)	6.900 (2.751-15.305)	< 0.001	7.149 (2.654-19.258)	< 0.001	6.769 (2.142-21.387)	0.001	7.419 (2.122-25.935)	0.002
SHR2 (per 0.1-point increase)	1.539 (1.280-1.849)	< 0.001	1.636 (1.326-2.020)	< 0.001	1.554 (1.222-1.974)	< 0.001	1.636 (1.250-2.140)	< 0.001

Abbreviations: SHR Stress hyperglycemia ratio, SHR1 was defined as [RBG (mmol/L)]/[(1.59*HbA1c) - 2.59] and SHR2 was defined as [FBG (mmol/L)]/[(1.59*HbA1c) - 2.59].

Model 1 adjusted for age and sex.

Model 2 adjusted for age, sex, current smoking, hyperlipidemia, atrial fibrillation, prior stroke, systolic blood pressure (SBP) and NIHSS at admission.

Model 3 adjusted for covariates from Model 2 and further adjusted for body mass index (BMI), diabetes and triglyceride-glucose index (TyG).

Table S3. Univariate and multivariate logistic regression analysis for 3-month mortality.

Variables	Crude Model		Model 1		Model 2		Model 3	
	OR (95% CI)	*p* value	OR (95% CI)	*p* value	OR (95% CI)	*p* value	OR (95% CI)	*p* value
SHR1 Q1 (< 1.02)	Ref.		Ref.		Ref.		Ref.	
SHR1 Q2 (1.02-1.15)	2.100 (0.479-9.213)	0.325	1.027 (0.202-5.224)	0.974	2.427 (0.306-19.271)	0.402	2.577 (0.301-22.086)	0.388
SHR1 Q3 (1.15-1.44)	3.062 (0.752-12.456)	0.118	2.202 (0.506-9.579)	0.293	6.136 (0.774-48.621)	0.086	7.446 (0.854-64.943)	0.069
SHR1 Q4 (> 1.44)	4.773 (1.242-18.345)	0.023	3.258 (0.775-13.693)	0.107	6.177 (0.869-43.924)	0.069	8.210 (1.007-66.972)	0.049
SHR1 (> 1.35 versus ≤ 1.35)	2.373 (1.013-5.555)	0.047	2.165 (0.843-5.561)	0.109	2.282 (0.683-7.622)	0.180	2.443 (0.650-9.186)	0.186
SHR1 (per 0.1-point increase)	1.129 (1.005-1.269)	0.041	1.115 (0.976-1.274)	0.110	1.141 (0.960-1.356)	0.135	1.174 (0.960-1.437)	0.118
SHR2 Q1 (< 0.79)	Ref.		Ref.		Ref.		Ref.	
SHR2 Q2 (0.79-0.89)	4.604 (0.499-42.473)	0.178	3.051 (0.311-29.904)	0.338	1.894 (0.163-21.969)	0.610	2.109 (0.173-25.694)	0.559
SHR2 Q3 (0.89-1.03)	9.957 (1.204-82.353)	0.033	7.035 (0.821-62.020)	0.075	4.187 (0.423-41.439)	0.221	5.153 (0.491-54.083)	0.172
SHR2 Q4 (> 1.03)	17.429 (2.183-139.159)	0.007	13.802 (1.645-115.779)	0.016	7.714 (0.794-74.903)	0.078	11.528 (1.027-129.453)	0.048
SHR2 (> 0.92 versus ≤ 0.92)	5.506 (2.144-14.657)	< 0.001	4.891 (1.753-13.647)	0.002	3.132 (0.908-10.809)	0.071	3.592 (0.950-13.578)	0.059
SHR2 (per 0.1-point increase)	1.321 (1.129-1.547)	0.001	1.326 (1.112-1.582)	0.002	1.229 (0.995-1.517)	0.056	1.448 (1.073-1.953)	0.016

Abbreviations: SHR Stress hyperglycemia ratio, SHR1 was defined as [RBG (mmol/L)]/[HbA1c (%)] and SHR2 was defined as [FBG (mmol/L)]/[HbA1c(%)].

Model 1 adjusted for age and sex.

Model 2 adjusted for age, sex, current smoking, hyperlipidemia, atrial fibrillation, prior stroke, systolic blood pressure (SBP) and NIHSS at admission.

Model 3 adjusted for covariates from Model 2 and further adjusted for body mass index (BMI), diabetes and triglyceride-glucose index (TyG).

Table S4. Univariate and multivariate logistic regression analysis for early neurological improvement (ENI).

Variables	Crude Model		Model 1		Model 2		Model 3	
	OR (95% CI)	*p* value	OR (95% CI)	*p* value	OR (95% CI)	*p* value	OR (95% CI)	*p* value
SHR1 Q1 (< 1.02)	Ref.		Ref.		Ref.		Ref.	
SHR1 Q2 (1.02-1.15)	0.600 (0.276-1.303)	0.197	0.607 (0.278-1.328)	0.212	0.639 (0.304-1.561)	0.372	0.662 (0.287-1.525)	0.332
SHR1 Q3 (1.15-1.44)	1.379 (0.663-2.868)	0.389	1.389 (0.666-2.895)	0.381	1.537 (0.715-3.281)	0.273	1.522 (0.701-3.307)	0.288
SHR1 Q4 (> 1.44)	0.735 (0.343-1.574)	0.428	0.739 (0.345-1.534)	0.436	0.718 (0.325-1.588)	0.413	0.756 (0.329-1.739)	0.511
SHR1 (> 1.35 versus ≤ 1.35)	0.972 (0.528-1.787)	0.926	0.972 (0.528-1.790)	0.928	0.909 (0.481-1.717)	0.768	0.914 (0.488-1.906)	0.964
SHR1 (per 0.1-point increase)	0.981 (0.900-1.068)	0.654	0.981 (0.900-1.069)	0.657	0.979 (0.897-1.069)	0.640	0.986 (0.895-1.085)	0.766
SHR2 Q1 (< 0.79)	Ref.		Ref.		Ref.		Ref.	
SHR2 Q2 (0.79-0.89)	0.560 (0.265-1.186)	0.130	0.563 (0.265-1.198)	0.119	0.575 (0.165-1.248)	0.161	0.576 (0.264-1.255)	0.165
SHR2 Q3 (0.89-1.03)	0.575 (0.271-1.219)	0.149	0.579 (0.271-1.237)	0.141	0.557 (0.150-1.243)	0.153	0.562 (0.249-1.269)	0.166
SHR2 Q4 (> 1.03)	0.624 (0.293-1.332)	0.223	0.628 (0.293-1.342)	0.200	0.614 (0.270-1.394)	0.224	0.657 (0.268-1.611)	0.359
SHR2 (> 0.92 versus ≤ 0.92)	0.664 (0.379-1.165)	0.153	0.669 (0.380-1.177)	0.163	0.609 (0.327-1.133)	0.117	0.610 (0.313-1.187)	0.146
SHR2 (per 0.1-point increase)	0.901 (0.787-1.032)	0.131	0.902 (0.788-1.033)	0.139	0.887 (0.766-1.026)	0.107	0.881 (0.745-1.043)	0.141

Abbreviations: SHR Stress hyperglycemia ratio, SHR1 was defined as [RBG (mmol/L)]/[HbA1c (%)] and SHR2 was defined as [FBG (mmol/L)]/[HbA1c(%)].

Model 1 adjusted for age and sex.

Model 2 adjusted for age, sex, current smoking, hyperlipidemia, atrial fibrillation, prior stroke, systolic blood pressure (SBP) and NIHSS at admission.

Model 3 adjusted for covariates from Model 2 and further adjusted for body mass index (BMI), diabetes and triglyceride-glucose index (TyG).

3 patients without 24h-NIHSS scores were excluded from the analysis.

Figure S1: Receiver operating characteristic (ROC) curves for admission random blood glucose (RBG), fasting blood glucose (FBG), and stress hyperglycemia ratio (SHR) with 3-month poor function outcomes as endpoint.

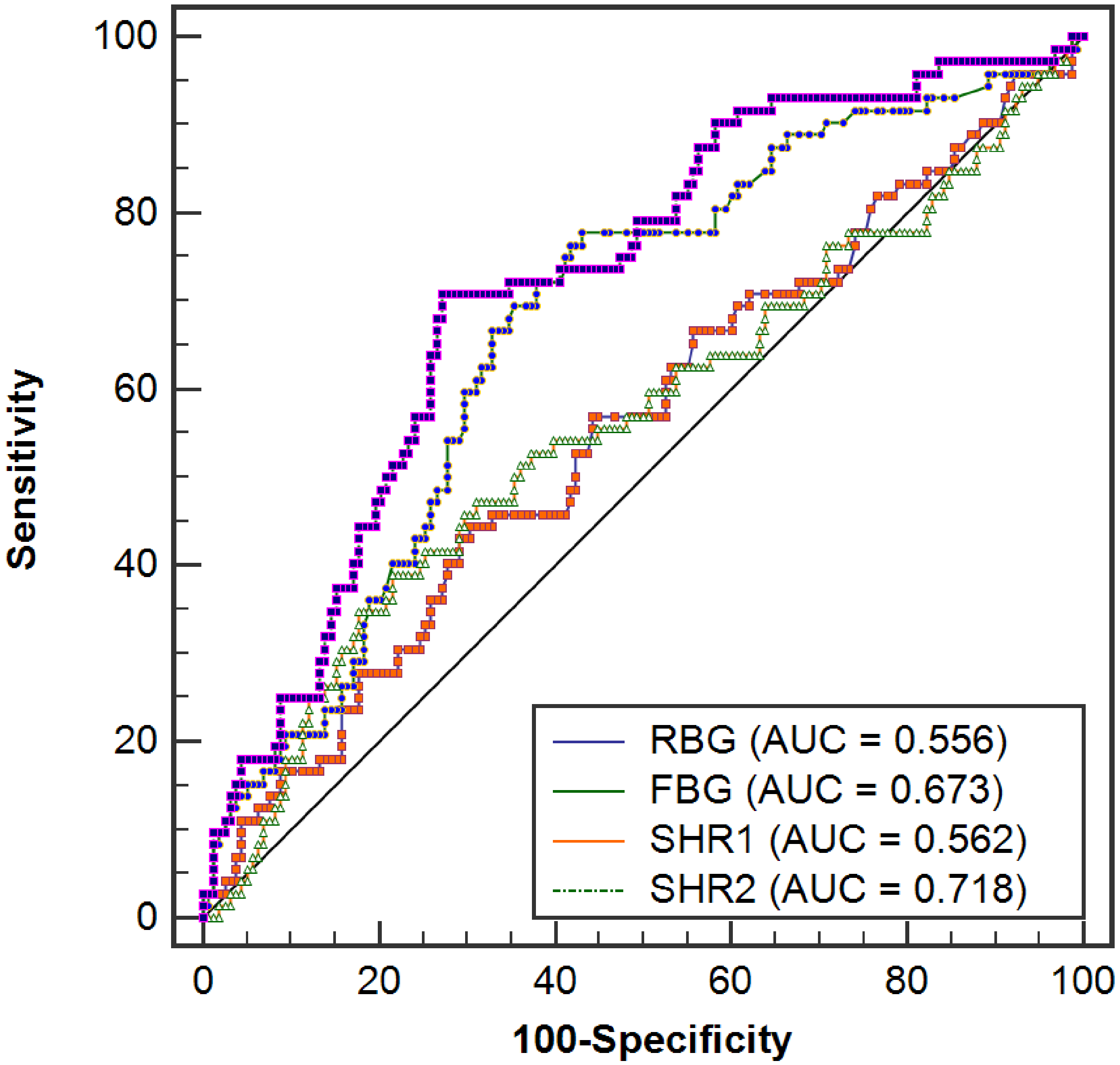

SHR1 was defined as [RBG (mmol/L)]/[HbA1c (%)] and SHR2 was defined as [FBG (mmol/L)]/[HbA1c(%)].
